# The influence of lifestyle and biological factors on semen variability

**DOI:** 10.1007/s10815-024-03030-y

**Published:** 2024-01-31

**Authors:** Hannah E. Lyons, Prabin Gyawali, Nicola Mathews, Patience Castleton, Shadrack M. Mutuku, Nicole O. McPherson

**Affiliations:** 1 Robinson Research Institute, The University of Adelaide, Adelaide, SA 5005 Australia; 2 Freemasons Centre for Male Health and Wellbeing, The University of Adelaide, Adelaide, SA 5005 Australia; 3 Discipline of Reproduction and Development, School of Biomedicine, Adelaide Medical School, The University of Adelaide, Level 5, Adelaide Health and Medical Sciences Building, Adelaide, SA 5000 Australia; 4 Repromed, 180 Fullarton Road, Dulwich, South Australia 5065 Australia

**Keywords:** Sperm, Infertility, Fertility, Body mass index, Nutrition, Exercise

## Abstract

**Purpose:**

Semen parameters are subjected to within-individual variability over time. The driving factors for this variability are likely multi-factorial, with healthier lifestyle associated with better semen quality. The extent in which variations in individual’s lifestyle contributes to within-individual semen variability is unknown.

**Methods:**

A total of 116 repeat semen samples from 29 men aged 19–37 over 6 months were collected. Basic semen analysis as per 5th WHO manual and extended semen parameters (sperm DNA fragmentation, redox potential and lipid peroxidation, sperm binding to hyaluronan and hyperactive motility) were assessed. An additional 39 lifestyle/biological factors (weight, blood pressure, etc.) were collected at each sample including validated health questionnaires SF36 Health Status, Australian Recommend Food Score, and International Physical Activity Questionnaire.

**Results:**

Only 10 out of the 39 lifestyle factors varied within men across samples including age (*P* = *0.0024*), systolic blood pressure (*P* = *0.0080*), social functioning (*P* = *0.0340*), energy (*P* = *0.0069*), non-alcoholic caffeinated beverages (*P* = *0.0010*), and nutrition (*P* < *0.0001*). The only semen parameter that varied between collections was sperm morphology (coefficient of variation 23.8 (6.1–72.0), *P* < *0.05*). We only observed weak (*r* < *0.3*) to moderate (*r* > *0.3*– < *0.6*) correlations between lifestyle factors, including body mass index, waist circumference, nutrition, exercise, blood pressure and semen parameters including sperm count, progressive motility, and sperm DNA fragmentation (*P* < *0.05*).

**Conclusion:**

In healthy men from the general population, semen quality and associated lifestyle factors do not significantly vary over 6 months, indicating that one semen sample is likely sufficient for determining male fertility in this population.

**Supplementary Information:**

The online version contains supplementary material available at 10.1007/s10815-024-03030-y.

## Introduction

It is known that semen parameters are subjected to within-individual variability over time, which has resulted in the recommendation of at least two semen samples for use in the diagnosis of male infertility [1]. The main reason is to decrease the false error rate in classifying men as fertile, subfertile, or infertile, with some even recommending that three semen samples should be collected due to this variability in semen quality [2]. Additionally, there is some evidence that semen variability within men is maybe more related to their fertility status, with healthy individuals showing less variability over time compared with subfertile men [2–4]. However, the real-world clinical application of performing repeat semen samples for diagnosing fertility status in men from the general population is likely low [5].

The driving factors in semen variability over time within the same man are likely multi-factorial, although we know that modifications in an individual’s lifestyle and biological factors (weight, exercise, nutrition, health and emotional status, metabolic and cardiovascular function, etc.) likely play a part. For instance, we know that dietary composition with increased intake of fruits and vegetables and reduced intake of sugar, processed red meats, and dairy products is associated with better sperm counts, motility, and morphology [6, 7]. Decreased physical activity (moderate-to-vigorous activity (< 5 h per week)) is associated with a 73% lower sperm count compared with men who perform > 15 h of moderate-to-vigorous physical activity per week [8]. Men with hypertension have lower sperm motility and total sperm counts due to a reduced semen volume [9]. However, the extent in which variations in an individual’s lifestyle and biological factors contribute to the variability of semen quality within men over time is unknown.

In this study, we collected four repeat semen samples over a period of 6 months in men from the general population, while at the same time recorded several anthropometric, lifestyle, and biological factors that have been linked with reduced semen quality. This was done so we could (1) better determine the within-individual variability of basic semen parameters (count, motility, and morphology) and extended semen parameters (hyperactivation, redox potential, sperm binding, DNA damage, and lipid peroxidation) to determine if more than one semen sample is really required to capture the fertility status of men from the general population and (2) understand the lifestyle and biological factors that might be driving semen variability within an individual as these factors could be prime targets for preconception health messaging.

## Materials and methods

### Ethics/participant recruitment

This project was approved by the Human Research Ethics Committee at the University of Adelaide (H-2020–163), and all protocols followed The National Statement on Ethical Conduct in Human Research (2007)—updated 2018. All participants provided informed consent and were reimbursed for their time. In total, 31 English speaking men between the age of 18 and 45 years were recruited from the general population (2020–2021), providing four semen samples over a 6-month period, at least 4 weeks apart (The ROSS cohort) [10]. Twenty-nine participants provided all four semen samples, while two participants provided only a single semen sample, and therefore were excluded from the study. For this study, only the samples where four repeated semen samples were collected were included, equating to a total of 116 samples from 29 participants. All samples were analysed at the Adelaide Health and Medical Sciences Building at the University of Adelaide. Exclusion criteria were men with a history of vasectomy or vasectomy reversal, men with undescended testicles or genetic conditions affecting their fertility (i.e. Prader-Willi and Klinefelter’s syndrome), and men with known infectious status, such as HIV/AIDS.

### Point of care

#### Height

The participants height was measured using a portable height measure rod. The measuring rod was stabilised by being positioned against a wall for maximum accuracy. Participants were then instructed to remove shoes, stand on the feet outlined on the base of the height measure rod, facing away from the rod while looking straight ahead. The headpiece was slid down the rod until it touched the top of the head of the participant. The measurement aligning with the height marker (in meters) attached to the headpiece was recorded.

#### Weight

A digital bathroom scale (Shekel Scales, Hamerkava, Israel) was used to measure weight in kilograms. Participants were instructed to remove shoes, heavy outer clothing and accessories, and heavy objects in their pockets prior to the measurement. Measurement was taken twice for accuracy and averaged.

#### Body mass index (BMI)

BMI was calculated using the Quetelet index (weight in kilograms (kg) and height in meters (m)) and the standard used by the Commonwealth Scientific and Industrial Research Organisation (CSIRO) to measure BMI for Caucasian adults aged 18 years and over [11]:

1. Underweight (BMI < 18.5 kg/m^2^)
2. Healthy weight (BMI ≥ 18.5 and BMI < 24.9 kg/m^2^)
3. Overweight (BMI ≥ 25 and BMI < 29.9 kg/m^2^)
4. Obese (BMI ≥ 30 kg/m^2^)

#### Abdominal circumference

Waist circumference was measured using a standard measuring tape set around the waist at a level midway between the lower rib and the iliac crest. Participants were instructed to fully exhale, with the abdomen relaxed.

#### Blood pressure measurement

Participants’ blood pressure (BP) was measured using an automatic BP cuff (GE Medical Systems, Buckinghamshire, UK) after being asked to sit and relax for 3–5 min. Literature shows that an individual’s BP is the highest when they first enter a clinic [12]; therefore, two BP measures were taken and the second was recorded. Systolic and diastolic measures were recorded in millimetres of mercury (mmHg). High BP, often associated with high stress levels and obesity, was classified as a reading ≥ 140/90 mmHg according to the WHO [13].

#### Glucose, cholesterol, and triglycerides

Whole blood samples were taken from a finger-prick, and blood glucose levels were tested using a point of care whole blood glucose meter (Accu-Chek Performa Nano, Roche, Laval, Quebec) and a glucose strip (Accu-Chek Performa glucose strips, Roche) while total cholesterol and triglycerides (Accutrend Plus cholesterol strips, Roche) were measured on a (Accutrend Plus Cobas, Roche). At the collection, it was noted whether the participant had fasted (≥ 8 h, no food, tea, or coffee) prior to the measurement.

### Healthy lifestyle questionnaires

On the day of each semen collection, participants were required to complete a healthy lifestyle questionnaire via our secure online portal, REDCap (Research Electronic Data Capture). These questionnaires included demographics/health and wellbeing data, reproductive histories, socioeconomic status, and education level as well as questions relating to lifestyle factors that have been shown to influence semen parameters such as smoking status, alcohol intake (units and days per week), and current medications (prescription and supplements). The sample questionnaires took approximately 15–25 min and included the following verified questionnaires.

#### Berlin Sleep Apnoea Questionnaire

The Berlin Sleep Apnoea Questionnaire is a validated patient survey that helps to identify patients at high risk of obstructive sleep apnoea (OSA) and to identify those snoring patients who have a low risk for OSA [14]. It assesses three OSA risk categories: (i) the presence and frequency of snoring behaviour, (ii) wake time sleepiness or fatigue, and (iii) a history of obesity and/or hypertension. Depending on the scores of these categories, participants are determined either high or low risk for OSA. The scoring criteria for each question/category can be seen in Supplementary Table 1. If ≥ 2 categories of the 3 are positive, the participant is at high risk of OSA, and if there is ≤ 1 category with a positive score, the participant is at low risk of OSA.

#### SF36 Health Status Survey

The Short Form 36 Health Status Survey developed at RAND as a part of the Medical Outcomes Study comprises a set of generic, coherent, and easily administered quality-of-life measures, separated into eight categories to measure aspects of physical functioning (10 questions), physical health (4 questions), emotional functioning (3 questions), energy/fatigue (4 questions), emotional wellbeing (5 questions), social functioning (2/1 question/s), pain (2 questions), and general health (4 questions) (Supplementary Table 2) [15]. Answers were recoded to a scoring system that ranges from 0 to 100. A higher score represents a more favourable health score. These recorded values are averaged to create a score for each separate category (Supplementary Table 2).

#### Australian Recommend Food Score (ARFS)

The ARFS is a series of questions regarding types of food groups recommended by the Australian Dietary Guidelines [16]. There are 22 possible points relating to vegetable intake, 12 relating to fruit, seven to meat, six to vegetable protein, 13 to bread/cereals/grains, 11 to dairy, and two to spreads/sauces, with a total possible score ranging from 0 to 73. Some foods were awarded an additional point if they were consumed more than once per week, while others were awarded an additional point if they were consumed more than once per week up to an upper threshold. Additional points were given where a greater number of vegetables, or healthier types of breads and milk were consumed. The scoring criteria used for each question/category can be seen in Supplementary Table 3. The higher the food score, the healthier the diet.

#### International Physical Activity Questionnaire (IPAQ)

The International Physical Activity Questionnaire was used to evaluate health-related physical activity of participants [17]. It asks a series of questions relating to frequency (days per week), duration (mins per week), and intensity (walking, moderate, vigorous) of exercise performed in the past 7 days. Participants are allocated into one of three categories based on their exercise patterns over the last 7 days: category 1, inactive; category 2, moderate activity; and category 3, high activity. The flow chart used to categories participants is detailed in Supplementary Fig. 1.

### Semen measures

#### Semen analysis

Participants were asked to abstain from ejaculation for 2–7 days prior to semen collection. Semen samples were produced in either one of the private, clinical rooms in the University of Adelaide’s Clinical Research Facility and collected in a sterile container, or produced at home and brought in within 45 min of ejaculation. Only approved lubricant (Ovoil, Vitrolife, Goteborg, Sweden) was used in the study at participant request. Participants who produced at home were provided with a semen collection pack and detailed instructions for how to correctly collect a semen sample at home, including correct room temperature transport requirements. Men who could not transport their semen sample to the laboratory within 45 min of ejaculation had to produce on site. After liquefaction at room temperature, a standard semen analysis was performed within 1 h of ejaculation as per WHO V guidelines for the assessment of human semen [18]. Semen volume was measured using a 10-mL serological pipette and semen pH measured using pH strip indicators ranging from pH 4.5 to 10 (Merck, Darmstadt, Germany). Sperm concentration and motility were measured on the CASA® semi-automatic semen analyser (Microptic, Spain, Barcelona), where at least 500 sperm were counted across a minimum of five fields of view. Low- and high-quality control beads (Microptic) were run prior to each sample analysis. A pre-set human count/motility program was used to calculate sperm concentration (10^6^/mL), total count (10^6^/ejaculate), and proportion of progressive (STR > 80% (STR = straight linear velocity/average path velocity*100)), non-progressive (STR < 80%), and immotile sperm. Total motility was calculated by the proportion of progressive and non-progressive sperm. Sperm morphology was assessed after 10 µL semen smears were made on glass slides and fixed in 100% methanol for 10 min. Diff-Quik® (RAL Diagnostic, Martillac, France) stain was then applied and sperm morphology assessed under 60 × objective with 200 sperm classified as either abnormal or normal morphology according to the Kruger strict criteria [18]. The proportion (expressed as %) of sperm with normal morphology was then calculated.

#### Sperm motility kinetics

Sperm motility kinetics were assessed following a swim up in G-IVF PLUS (Vitrolife) in motile sperm fractions (> 95% progressive motility) on the CASA® semi-automatic system (Microptic) collected as an extension of the motility program. A total of 500 sperm were counted and motility kinetics of sperm curvilinear velocity (m/s) (VCL), straight-line velocity (km/s) (VSL), average path velocity (km/s) (VAP), amplitude of lateral head displacement (km) (ALH), linearity (%) (LIN), wobble (%) (WOB), straightness (%) (STR), and beat-cross frequency (Hz) (BCF) were automatically calculated via the CASA® system. The percentage of sperm hyperactivation was calculated via the CASA® system based on the proportion of sperm with sperm motility kinetics amplitude of lateral head displacement > 3.5 µM, curvilinear velocity > 80 µM/s, and linearity < 20% (straight line velocity/curvilinear velocity × 100).

#### Sperm binding—HBA® hyaluronan binding assay

Sperm binding was assessed using the HBA® hyaluronan binding assay (CooperSurgical Fertility Solutions, Knardrupvej, Denmark). Briefly, 10 µL of semen was loaded into the assay chamber and a Cell-Vu® gridded coverslip was installed. The sample was incubated for 15 min at 20–30 °C. A total of 200 motile sperm were then counted and classified as either bound (head attached and tails moving) or unbound (freely moving). Scores of 80% or higher were classified as normal binding and those below this threshold were classified as displaying reduced binding as stipulated by the manufacture.

#### Redox potential: MiOXSYS® system

The MiOXSYS® system (Aytu Bioscience, Colorado, USA) was used to detect total static oxidative-reductive potential (sORP), or redox potential, within a semen sample as per manufacturer’s instructions [20]. The sORP (mV) value for samples was automatically generated on the MiOXSYS® reader. High and low controls supplied separately by the manufacturer were run monthly, ensuring consistency in machine detection, with the lowest detectable limit being 0.001 sORP (mV).

#### Sperm DNA fragmentation: HALOSPERM G2®

Sperm DNA fragmentation was measured using Halosperm G2® (Halotech DNA, Madrid, Spain) as previously described by Fernandez et al., [21], and visualised and assessed using the CASA® semi-automatic semen analyser (Microptic) under the pre-set human sperm DNA fragmentation program. Sperm were classified as having small (smaller than 1/3 diameter of the nuclei core), medium (150–170 µm^2^), or large (250–280 µm^2^) halos, as well as degraded or absent halos. Sperm DNA fragmentation index (DFI) percentage was calculated with the following equation: (fragmented + degraded / 200) × 100, in which fragmented DNA included small and absent halos. A pooled control comprised of 16 randomly selected participant samples was stained and analysed at the time of each test to ensure consistency within the assay and reagents; a maximum standard error of 3% was considered an acceptable range.

#### Lipid peroxidation—BODIPY™ 581/591 C11

Motile sperm (1 × 10^6^/mL, > 95% progressive motility) collected following a swim up in G-IVF PLUS (Vitrolife) were incubated in 5 µM of BODIPY 581/591 fluorescent probe for 30 min at 37 °C, as previously described by Aitken et al. [22]. This reagent localises to membranes throughout live cells and upon oxidation by lipid hydroperoxides, displays a shift in peak fluorescence emission from ∼590 nm (red) to ∼510 nm (green). Sperm were then centrifuged at 400 × g for 5 min, the supernatant was removed, and sperm were resuspended in pre-equilibrated (37 °C, 5% O_2_ and 6% CO_2_) G-IVF minus albumin (Vitrolife, Sweden). Lipid peroxidation was assessed on a BD FACSCanto™ II Flow Cytometer (BD Bioscience, NSW, Australia), which had CST beads run daily to ensure fluorescence was kept consistent on measurement days. Then, 10,000 cells per sample were examined and non-specific events gated out. Positive controls of 3000 µM of hydrogen peroxide spiked preparation were run monthly. Negative controls consisted of sperm incubated in G-IVF medium alone. Lipid peroxidation was expressed as the proportion of sperm that had high lipid peroxidation [22]. Details of our gating strategy are presented in our previous publication [10].

### Statistics

GraphPad Prism v9.0 for Windows (GraphPad Software, San Diego, CA, USA, www.graphpad.com) and IBM SPSS Statistics for Windows, version 26 (IBM Corp., New York, USA) were used for all statistical analyses. All basic and extended semen parameters were transformed by natural logarithm to normalise data. To determine the variation in biological and lifestyle factors as well as basic and extended semen parameters, data was analysed by a repeated measures ANOVA with Tukey’s multiple comparisons test. The intra-subject coefficient of variation (CV_w_) was calculated by the standard deviation divided by the mean and multiplied by 100. The closer to 100, the higher the variation within an individual. Correlations between biological and lifestyle factors and measure of semen quality were assessed through a Pearson’s correlation with outcomes displayed as the Pearson’s *r* coefficient. In all cases, statistical significance was inferred when *P* < *0.05*.

## Results

### Participant demographics

Table 1 shows the demographic and reproductive history data of men in our study. Majority of men were of Caucasian decent (24/29, 82.8%), with a university or college degree (18/29, 62.1%) between the ages of 19–37 years. Six of the men (20.7%) had previously fathered a pregnancy and 15 men were in a long-term relationship (51.7%). All 29 men provided four semen samples and completed all associated anthropological measures and lifestyle questionnaires at each collection.

**Table 1:** Participant characteristics

	Number (%)
Country of birth	
Australia	22 (75)
India	1 (3.4)
Iran	1 (3.4)
Malaysia	2 (6.9)
Mongolia	1 (3.4)
UK or Ireland	2 (6.9)
Highest qualification	
High school	9 (31.0)
University or college degree	18 62.1)
Trade, technical certificate, diploma	2 (6.9)
Work status	
Full time	11 (37.9)
Part time/casual	6 (20.7)
Student	10 (34.5)
Unemployed	2 (6.9)
Yearly income (AUD)	
< $12 K	6 (20.7)
$12–20 K	2 (6.9)
$20–30 K	5 (17.2)
$30–40 K	2 (6.9)
$40–50 K	2 (6.9)
$50–60 K	1 (3.4)
$60–80 K	5 (17.2)
> $80 K	6 (20.7)
Shift worker	
Yes	3 (10.3)
No	26 (89.7)
Relationship status	
Single	13 (44.8)
De facto	5 (17.2)
Married	6 (20.7)
Partner/spouse	4 (13.8)
Rather not say	1 (3.4)
Prescription/non-prescription medication	
Yes	11 (37.9)
No	18 (62.1)
Smoker (tobacco)	
Yes	1 (3.4)
No	28 (96.6)
Obstructive sleep apnoea	
High risk	2 (6.9)
Low risk	27 (92.1)
Snore (yes)	9 (31.0)
Previous pregnancy	
Yes	6 (20.7)
No	23 (79.3)
Previous reproductive surgery	
Yes	3 (10.3)
No	26 (89/7)
Testicular injury	
Yes	1 (3.4)
No	28 (96.6)
Pain passing urine	
Yes	3 (10.3)
No	26 (89.4)
Previous sexually transmitted infection	
Yes	1 (3.4)
No	28 (96.6)
Exposure to toxic chemicals	
Yes	2 (6.9)
No	27 (92.1)
Exposure to radiation	
Yes	0 (0)
No	29 (100)
Hot working conditions	
Yes	4 (13.8)
No	25 (86.2)

### Variation in lifestyle factors in men over 6 months

Table 2 shows the variation in measured lifestyle and biological factors within individual participants. Across the 39 factors assessed, we only observed significant variations in ten factors across the four repeated collections, which included age (*P* = *0.0024*), systolic blood pressure (*P* = *0.0080*), social functioning (*P* = *0.0340*), energy (*P* = *0.0069*), number of non-alcoholic caffeinated beverages (*P* = *0.0010*), and total food score (*P* < *0.0001*) (with differences in scores for bread/cereal, fruits, dairy, and spreads *P* < *0.01*).

**Table 2:** Biological and lifestyle variability over 6 months

	Sample collection				
	1st	2nd	3rd	4th	Repeated measure ANOVA (*P*-value)
Point of care					
Age (years)	25 (19–37)^a^	25 (19–37)^a^	26 (19–37)^ab^	26 (19–37)^b^	***0.0024***
Weight (kg)	76.0 (56.7–111.8)	76.9 (58–113.2)	77.1 (59.0–115.4)	76.8 (60.2–114.6)	*0.8193*
Waist circumference (cm)	82.0 (67.7–112.3)	82.2 (66.0–110.6)	84.3 (67.9–114.6)	82.0 (69.2–111.1)	*0.2394*
Body mass index (kg/m^2^)	24.1 (19.0–33.5)	24.4 (19.4–32.8)	24.6 (20.1–31.9)	24.6 (19.8–32.7)	*0.7053*
Systolic blood pressure (mmHg)	128 (99–159)^a^	122 (105–140)^ab^	121 (104–147)^b^	122 (106–144)^b^	***0.0080***
Diastolic blood pressure (mmHg)	76 (49–114)	73 (48–96)	71 (52–96)	74 (54–96)	*0.0978*
Pulse rate (beats per minute)	80 (51–108)	78 (50–108)	77 (51–99)	77 (52–95)	*0.3708*
Fasted blood sample	9/29 (31%)	6/29 (21%)	6/29 (21%)	6/29 (21%)	*-*
Glucose (mmol/L^−1^)	5.6 (4.6–8.5)	5.6 (1.2–7.3)	5.6 (4.3–7.0)	5.4 (4.6–7.8)	*0.6999*
Cholesterol (mmol/L^−1^)	3.9 (3.8–5.7)	3.9 (3.9–5.2)	3.9 (3.8–5.5)	3.9 (3.8–6.7)	*0.9730*
Triglycerides (mmol/L^−1^)	0.7 (0.7–3.8)	0.7 (0.7–2.1)	0.7 (0.7–3.1)	0.7 (0.7–4.3)	*0.4096*
Health questionnaires					
*SP-30 Health status*					
General health	75 (38–100)	75 (38–100)	75 (38–100)	75 (25–100)	*0.1414*
Physical functioning	93 (5–100)	94 (0–100)	89 (0–100)	94 (0–100)	*0.6839*
Physical health	100 (25–100)	100 (0–100)	100 (25–100)	100 (0–100)	*0.7707*
Emotional problems	100 (0–100)	100 (0–100)	100 (0–100)	100 (0–100)	*0.8532*
Social functioning	100 (50–100)	88 (30–100)	76 (13–100)	88 (20–100)	***0.0340***
Emotional wellbeing	76 (32–92)	76 (32–92)	72 (20–92)	72 (28–92)	*0.4978*
Energy	60 (15–85)^a^	55 (15–85)^ab^	55 (15–85)^b^	60 (25–85)^ab^	***0.0069***
Average pain	90 (45–100)	90 (58–100)	90 (45–100)	90 (45–100)	*0.3885*
*Australian food frequency*					
Total score	44 (18–59)^a^	37 (17–51)^b^	38 (12–64)^b^	39 (18–56)^b^	** < *0.0001***
Breads/cereals	6 (1–10)^a^	5 (2–7)^b^	5 (2–9)^ab^	5 (2–9)^ab^	***0.0070***
Fruit	9 (1–12)^a^	7 (1–12)^ab^	7 (0–12)^b^	7 (0–11)^ab^	***0.0069***
Vegetable	19 (4–23)	16 (4–22)	17 (4–22)	17 (3–23)	*0.0757*
Dairy	3 (0–5)^a^	1 (0–3)^b^	1 (0–4)^b^	1 (0–4)^b^	** < *0.0001***
Spreads	2 (1–2)^a^	1 (0–2)^b^	1 (0–2)^b^	1 (0–2)^b^	***0.0154***
Meat	4 (0–6)	3 (0–6)	4 (0–6)	4 (0–6)	*0.1349*
Vegan protein	4 (1–6)	4 (1–6)	4 (1–6)	4 (1–6)	*0.1903*
*Alcohol frequency*					
Alcohol consumption (days/week)	1.0 (0.0–3.5)	1.0 (0.0–3.5)	1.0 (0.0–15.0)	1.0 (0.0–3.5)	*0.2425*
Alcohol consumption (units/week)	2.3 (0.0–10.5)	2.3 (0.0–10.5)	2.3 (0.0–10.5)	1.5 (0–10.5)	*0.2078*
Non-alcoholic caffeinated beverages (units/day)	3 (1–4)^a^	1 (1–3)^b^	2 (1–4)^b^	2 (1–4)^b^	***0.0010***
*International Physical Activity Questionnaire*					
Overall score	2 (1–3)	2 (1–3)	2 (1–3)	2 (1–3)	*0.3702*
Days of vigorous physical activity (last 7 days)	3 (0–7)	3 (0–6)	2 (0–7)	2 (0–7)	*0.9062*
Time spend during vigorous physical activity (min) on one of those days	50 (0–240)	60 (0–240)	60 (0–300)	45 (0–90)	*0.0712*
Days of moderate physical activity (last 7 days)	3 (0–6)	3 (0–7)	3 (0–7)	3 (0–7)	*0.4202*
Time spend during moderate physical activity (min) on one of those days	20 (0–300)	30 (0–480)	30 (0–240)	20 (0–240)	*0.4740*
Days of walking (last 7 days)	7 (0–14)	6 (0–7)	6 (1–7)	7 (1–7)	*0.3871*
Time spend walking (min) on one of those days	45 (0–360)	30 (0–480)	30 (5–435)	25 (3–497)	*0.8289*
Time spent sitting (h) (last 7 days)	8 (2–450)	7 (2–330)	8 (4–150)	8 (0–120)	*0.7386*
Abstinence (days)	3 (0.5–8)	3 (0.5–7)	3 (0.5–8)	3 (1–10)	*0.3212*

Data is expressed as median (range) for continuous data and percentages for proportional data. *N* = 116 semen samples from *N* = 29 men. Data was analysed by a repeated measures ANOVA with Tukey’s multiple comparisons test. Different symbols within the same row indicate significance (*P* < 0.05) between collections times

### Variation in semen quality in men over 6 months

Of the 29 individuals, 20 men (69.0%) returned an abnormal basic semen analysis (count, motility, morphology) result in at least one of their semen collections, with the remaining nine participants having consistent basic semen analysis reports above the WHO V reference ranges for all four samples. For extended semen parameters, eight men (27.6%) had at least one sample with DNA fragmentation concentrations ≥ 20%, 25 men (86.2%) had at least one sample with sperm binding to hyaluronan < 80%, 18 men (62.0%) had at least one sample with hyperactive sperm motility < 10%, and 12 men (41.4%) had at least one sample with sperm lipid peroxidation concentrations > 40%.

**Fig. 1:**
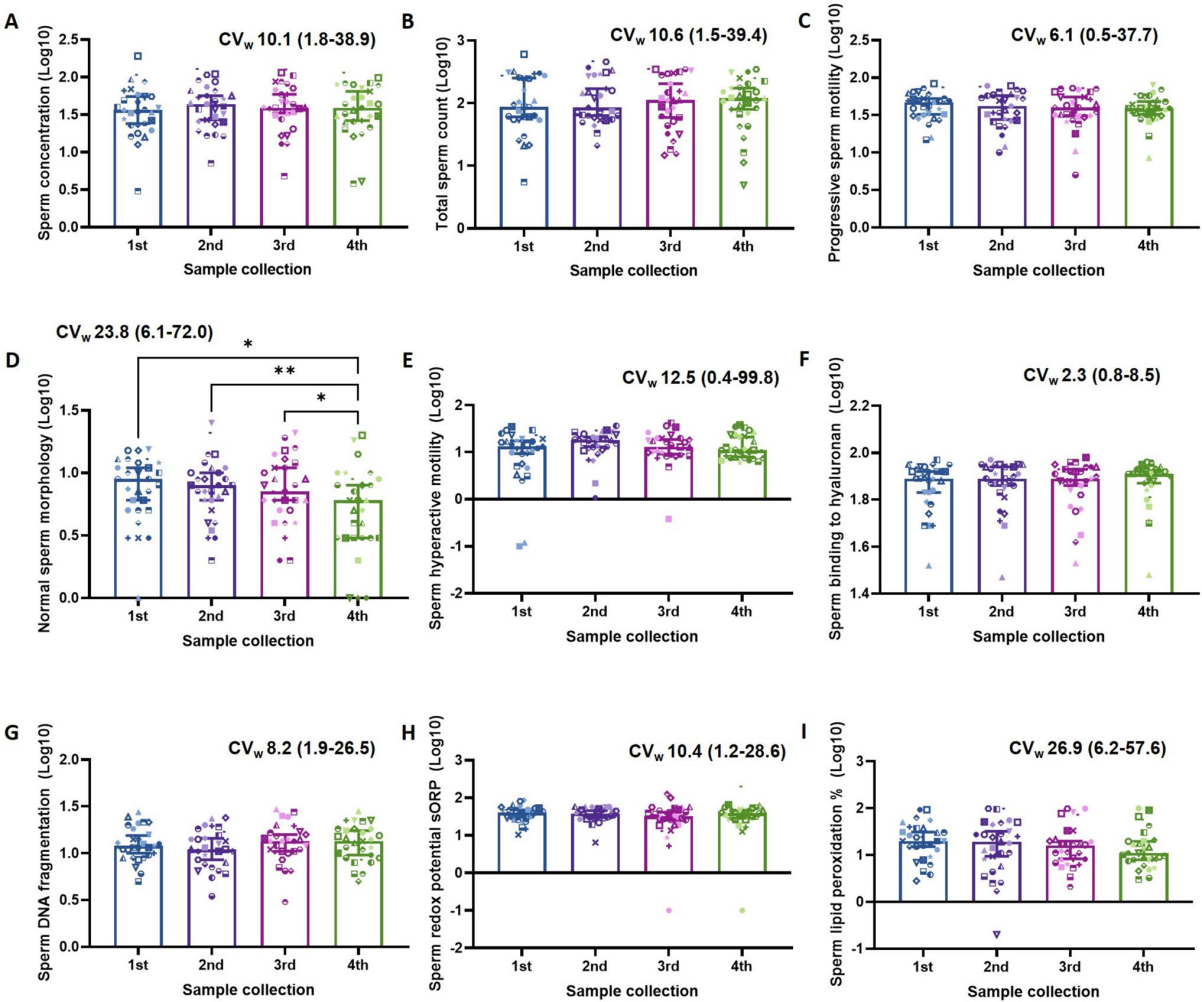
Semen parameters in men from the general population have minimal variability between repeat collections over 6-months. **A** Sperm concentration 10^6^/ml, **B** total sperm count 10^6^/ejaculate, **C** proportion of progressively motile sperm, **D** proportion of sperm with normal morphology, **E** proportion of sperm with hyperactive motility patterns, **F** proportion of motile sperm bound to hyaluronan, **G** proportion of sperm with DNA fragmentation, **H** semen redox potential sORP, and **I** proportion of sperm with high lipid peroxidation. Data is expressed as median with 95% confidence intervals and intra-subject coefficient of variation (CV_w_) with range. Data was analysed by a repeated measures ANOVA with Tukey’s multiple comparisons test. *N* = 116 semen samples from *N* = 29 men. *P < 0.05, **P < 0.01

When analysing the variability in both basic and extended semen parameters across the four samples (Fig. 1), surprisingly, the only parameter that showed significant variation between collections was sperm morphology (*P* < *0.05*, Fig. 1D). We further assessed this variability in those 20 participants that returned at least one abnormal basic semen parameter in any collection (Table 3) as there is some evidence that the variation would be greater in subfertile men [2, 3]. Yet again, the only parameter that showed a significant variation between collections was sperm morphology (*P* < *0.05,* Table 3).

**Table 3:** Semen parameters in men with at least one abnormal semen parameter in any collection have minimal variability between repeat collections over 6 months

	Semen collection				
	1st	2nd	3rd	4th	Repeated measure ANOVA (*P*-value)
Basic semen parameters					
Sperm concentration (Log10)	1.43 (0.48–1.84)	1.52 (0.85–1.84)	1.55 (0.68–2.02)	1.52 (0.58–1.92)	*0.4486*
Total sperm count (Log10)	1.79 (0.74–2.48)	1.84 (1.32–2.57)	1.99 (1.17–2.54)	2.07 (0.69–2.50)	*0.6812*
Progressive sperm motility (Log10)	1.60 (1.2–1.86)	1.53 (1.08–1.89)	1.60 (1.02–1.85)	1.56 (0.93–1.84)	*0.7775*
Normal sperm morphology (Log10)	0.82 (0.00–1.18)^ab^	0.83 (0.30–1.15)^a^	0.78 (0.30–1.20)^a^	0.65 (0.00–1.00)^b^	***0.0094***
Extended semen parameters					
Sperm hyperactive motility (Log10)	1.05 (− 1.00–1.54)	1.19 (0.03–1.56)	1.03 (− 0.42–1.620	1.06 (0.77–1.58)	*0.1339*
Sperm binding to hyaluronan (Log10)	1.89 (1.52–1.97)	1.88 (1.47–1.97)	1.89 (1.53–1.98)	1.91 (1.48–1.96)	*0.5859*
Sperm DNA Fragmentation (Log10)	1.09 (0.85–1.44)	1.10 (0.74–1.38)	1.15 (0.483–1.47)	1.16 (0.70–1.45)	*0.4639*
Semen redox potential sORP (Log10)	1.56 (1.02–1.94)	1.56 (0.81–1.77)	1.50 (0.72–2.00)	1.58 (1.10–1.82)	*0.2823*
% Sperm lipid peroxidation (Log10)	1.30 (0.45–1.97)	1.22 (− 0.70–1.98)	1.06 (0.32–1.99)	1.04 (0.48–2.00)	*0.4794*

Data is expressed as median with (range). *N* = 80 semen samples from *N* = 20 men. Data was analysed by a repeated measures ANOVA with Tukey’s multiple comparisons test. Different symbols within the same row indicate significance (*P* < 0.05) between collections times

We then looked to assess those men who returned an abnormal basic semen analysis (count, motility, or morphology) result from their initial semen collection to determine if this measure continued to be abnormal in subsequent collections. Eleven men (37.9%) returned an abnormal basic semen analysis result from their initial sample. We observed that there was a 67% concordance that any subsequent semen samples from that individual repeatedly returned the same abnormal result (Table 4).

**Table 4:** The likelihood of a basic semen parameter being below the lower reference limit in subsequent semen collections when the first initial semen collection is below the WHO reference limit

Participant	Measure below lower reference limit for first semen collection	# of subsequent semen collections below lower reference limit	% of concordance in subsequent collections
1	Progressive motility	3	100
2	Progressive motility	1	33
3	Progressive motility	2	67
4	Progressive motility Total sperm count	2 3	67 100
5	Total sperm count	2	67
6	Total sperm count	3	100
7	Total sperm count	0	0
8	Total sperm count	0	0
9	Normal morphology	3	100
10	Normal morphology	0	0
11	Normal morphology	3	100
Median			**67**

### The relationship between semen quality and biological and lifestyle factors

**Fig. 2:**
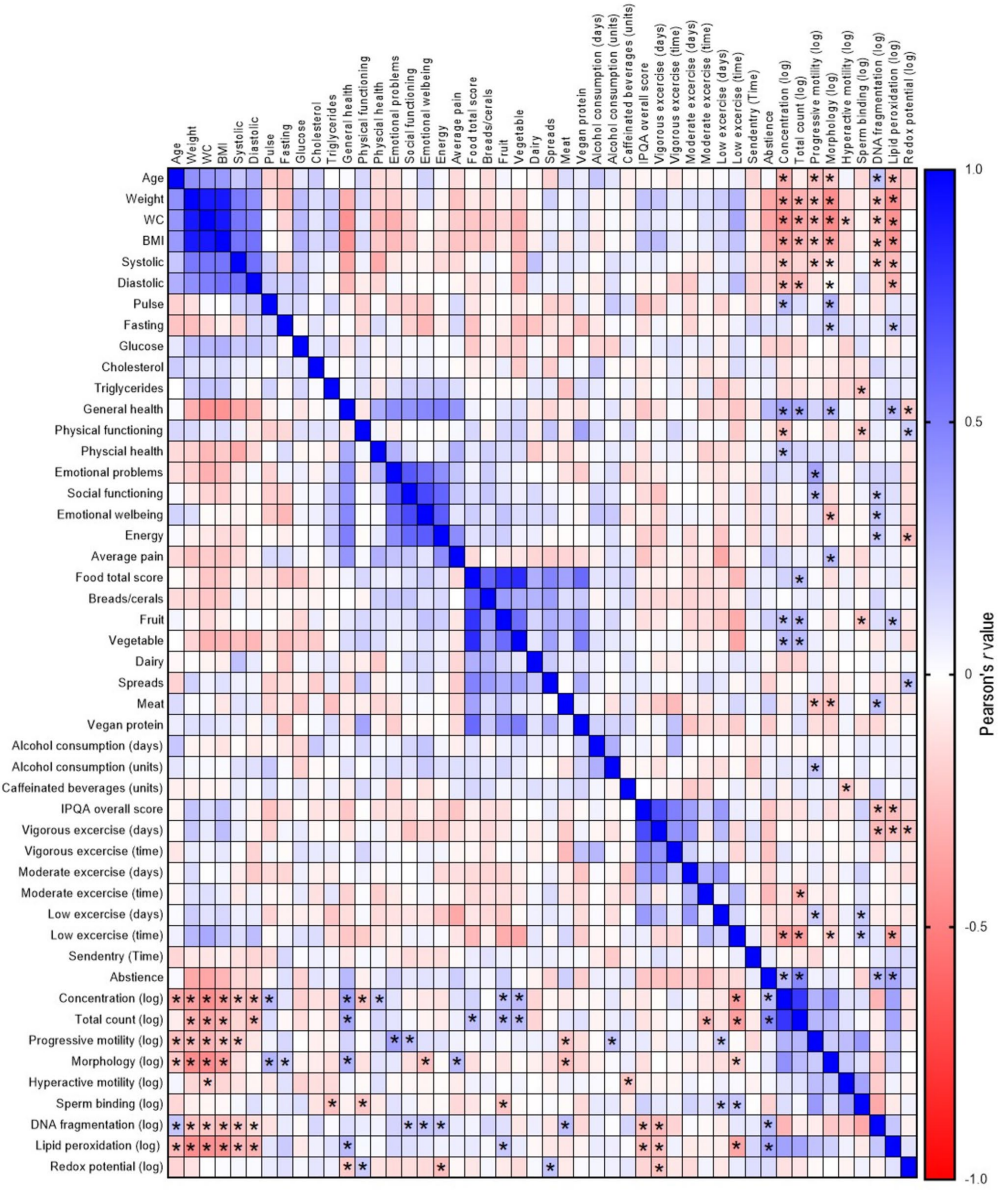
Heat map showing the correlation between semen parameters and biological and lifestyle factors. Data is expressed as the Pearson’s *r*-value with differing shades of blue showing positive associations and differing shades of red showing negative associations. *N* = 116 semen samples from *N* = 29 men analysed by a Pearson’s correlation. *P < 0.05

To gain insight into which lifestyle factors share relationships with sperm parameters, we performed correlation analysis displayed in Fig. 2. We only observed weak (*r* < *0.3*) to moderate (*r* > *0.3* and < *0.6*) correlations between measured lifestyle/biological factors and semen parameters. Age, weight, BMI, and waist circumference all had weak to moderate negative correlations (*r* >  *− 0.35*, *P* < *0.01*) with basic sperm parameter concentration, progressive motility, and morphology. We also observed weak positive and negative correlations with measure of health status and sperm quality, including physical functioning with sperm concentration (*r* =  *− 0.234*, *P* < *0.05*) and general health with sperm concentration, sperm morphology (*r* > *0.3*, *P* < *0.01*) and redox potential (*r* =  *− 0.21*, *P* < *0.05*). Dietary intake including intake of fruits and vegetables was moderately positively correlated with total sperm count (*r* > *0.3*, *P* < *0.01*), fruit intake weakly negatively correlated with sperm binding (*r* =  *− 0.22*, *P* < *0.05*) and increased meat consumption negatively associated with sperm progressive motility and morphology (*r* > *0.2*, *P* < *0.05*) and positively associated with sperm DNA fragmentation (*r* = *0.24*, *P* = *0.01*). IPQA score had weak negative correlations with sperm DNA fragmentation (*r* =  *− 0.23*, *P* < *0.05*) and lipid peroxidation (*r* =  *− 0.25*, *P* < *0.01*), while systolic and diastolic blood pressure had weak to moderate negative associations with sperm concentration, progressive motility, and morphology (*r* > *0.15*, *P* < *0.05*).

While the overall variation in biological and lifestyle factors was weak across the cohort, there were a small subset of participants (*N* = 3) who display close to a 3 kg/m^2^ change in BMI across the study period. Those men who displayed a change in BMI had a larger coefficient of variation in total sperm count across their repeated collections compared with those men who did not display a change in BMI (*P* < *0.01*, Supplementary Fig. 2).

## Discussion

Male fertility is currently assessed through a traditional semen analysis, which is often subjected to within-individual variation and the recommendation for repeat semen collections to confirm fertility status [23]. The rationale for this variation is likely due to differences in laboratory techniques and equipment, the duration of the subject’s abstinence period, seasonal changes, and modifications to biological and lifestyle factors. There are many lifestyle and biological factors that are associated with male fertility, including physical health, exercise, nutrition, and BMI [24], yet studies to date have only assessed their influence in isolation at one semen collection. To determine if modifications to an individual’s lifestyle could be contributing to the within-individual variation in semen quality over time, we collected four repeat semen samples and a wide variety of biological and lifestyle related measures at each semen collection in men from the general population. Interestingly, in our pilot trial, we observed very low variations in measured lifestyle and biological factors in men as well as both basic semen parameters (count, motility, and morphology) and extended semen parameters (sperm binding, hyperactive motility, DNA damage, redox status, and lipid peroxidation) over the 6 months.

Unexpectedly, across our pilot cohort of 29 men, only 10 out of the 39 lifestyle and biological factors measured showed significant variation across the repeat collections, with five of those 10 been reflective as changes in dietary intake. The variation in dietary intake was not surprising, as other have also reported that nutritional intake is one of the most variable lifestyle factors amongst men [25, 26]. The low level of variability in other lifestyle and biological factors measured is maybe reflective of the population recruited, who were young men in their prime reproductive years (18–40 years), of a higher socio-demographic and generally in good overall health (high general health scores, low BMI, non-smokers, etc.). These types of men are more likely to maintain healthier lifestyle behaviours over-time [27].

Interestingly, in contrast to other studies [2–4, 23], we observed very little variation in both basic and extended semen parameters across the 6-month period. This was evident in both normospermic men and in those men who displayed abnormal semen parameters. One rationale for our lack of within individual variation across collections could be due the minimal variation in biological and lifestyle factors described above, with no dramatic change in health status overtime equating too little change in semen quality overtime. This was further evident in those men whose BMI varied up to 2 kg/m^2^ displaying greater coefficient of variation in total sperm counts compared with men who maintained their BMI throughout the four collections. Another rationale for our reduced within-individual variation could be because majority of our semen measures were assessed using semi-automatic platforms (i.e. computer assisted semen analyser (CASA) and flow cytometry) removing the reported intra- and inter-technician variability in estimating semen outcomes [28]. The only measure that did show significant variation was that of sperm morphology, which was not assessed using a semi-automatic platform, rather a single technical operator. This further highlights that the reported within-individual semen variability overtime is likely more related to the intra- and inter-technical influence, and questions the removal of semi-automatic and automatic platforms for performing semen analysis in the latest 6th edition methods manual for the processing of human semen [1].

There is also evidence that the fertility status of men is maybe more related to semen variability, with normospermic men displaying reduced semen variability over time compared with subfertile men [2–4, 23]. In our study, when the initial sample collected returned an abnormal result, there was a 67% concordance that the remaining three subsequent samples would also return the same abnormal result. There were only three occasions where a participant returned an abnormal semen parameter in their first collection and then normal results on subsequent collections. These results align with that of Ursillo et al. [29], who also showed concordance in their second semen collection in approximately 75% of patients who returned an abnormal semen parameter (i.e. count and motility) on first collection. This suggests that the need for repeated semen samples for the diagnosis of male factor infertility due to a sperm defect may be unnecessary, as semen quality appears relatively stable across the time period routinely used for repeated semen analysis (~ 3–6 months).

Despite the lack of variability in semen parameters and biological and lifestyle factors across our pilot cohort, we still performed correlation analysis to determine if any one factor had greater associations with semen quality over time, as these factors could be key focal points in male preconception health messaging. We observed only weak to moderate associations likely due to our low sample size (a limitation of our pilot trial), which were consistent with those previously reported in the literature. For instance, we found negative associations between traditional sperm parameters of count, motility, and morphology with age [30], BMI [31, 32], waist circumference [33, 34], and general health and psychological wellbeing [35]. Dietary intake, which displayed the biggest variations across collections in our cohort, was also associated with semen quality. Increased intake of fruits and vegetables and overall total food score all showed positive correlations with total sperm count, while increased meat consumption was negatively associated with sperm motility and morphology and positively associated with sperm DNA fragmentation, findings that supports previous literature [6, 7]. In our study, we only observed minor relationships between measures of physical activity and basic semen parameters, with total IPAQ score and vigorous exercise days also negatively associated with extended semen parameters of sperm DNA damage and lipid peroxidation. There is still a debate in the literature as to the influence of physical activity on male fertility [36, 37] and how the type, duration, and intensity of exercise independently influences semen quality. In our study, we utilised the IPAQ to generate an overall physical activity score and to record the amount of time and number of days of sedentary, low, moderate, and high levels of exercise. However, this questionnaire did not report on the types of exercise performed, which is known to drive changes in semen quality [36, 37]. Another biological factor that showed significant variation across the repeated collections was systolic blood pressure. We observed negative associations between both diastolic and systolic blood pressure and basic semen parameters of concentration, total count, and progressive motility. There has been very little investigation into the influence blood pressure has on male fertility; however, these observations are in line with the reported differences in semen parameters between patients with hypertension compared with normotensive patients, where hypertensive men displayed significantly lower total sperm counts and motility [9].

The biggest limitation of our study was our sample size. We chose initially to perform a pilot trial to begin to decipher those biological and lifestyle factors driving semen variability within men over time in hopes to reduce the total numbers of measures we would have to record in a larger cohort. The low sample number (*N* = 118 samples from 29 men) also likely contributed to the weak to moderate strength associations between biological and lifestyle factors and measures of both basic and extended semen parameters, with some correlations going in the opposite direction to that expected. For instance, BMI was weakly negatively associated with sperm DNA damage and lipid peroxidation a direct opposite to that of the current literature [32]. Whilst other relationships correlated as expected, such as sperm DNA damage and lipid peroxidation both positively correlating with abstinence, a factor which is known to increase sperm oxidative stress [38]. Strengths of our study include the collection of four repeat semen samples from each of our participants, the assessment of extended semen quality including sperm binding to hyaluronan, sperm DNA fragmentation and lipid peroxidation, and the collection of over 35 independent lifestyle and biological factors at each collection point. Our data also provides the preliminary evidence that larger prospective cohorts are needed to further understand the most influential lifestyle and biological factors driving semen variability within men and to identify those key modifiable factors that can be primary targets of male preconception health messaging.

## Conclusion

In healthy men from the general population, semen quality and associated lifestyle and biological factors do not significantly vary over 6 months. Majority of the changes to within-individual semen variability are likely due to intra- and inter-technician differences or dramatic changes to one’s lifestyle and as such unless significant changes to either of those occur between sample collections, one semen sample is likely sufficient for determining male fertility status. While our study was not able to identify the lifestyle factors, which were the main drivers of semen quality variation over time, we were able to substantiate the relationship between certain key factors and sperm parameters, paving way for larger prospective cohorts.

### Supplementary Information

Below is the link to the electronic supplementary material.

Supplementary file1 (DOCX 402 KB)

## Data Availability

The data that supports the findings of this study are available from the corresponding author, NOM, upon reasonable request.
