# Supplementary material for: The influence of lifestyle and biological factors on semen variability
Source: J Assist Reprod Genet. 2024 Jan 31;41(4):1097–109. doi: 10.1007/s10815-024-03030-y (PMC11052966; doi:10.1007/s10815-024-03030-y)
Supplement: Supplementary file 1 — Supplementary file1 (DOCX 402 KB) [file 10815_2024_3030_MOESM1_ESM.docx]

**SUPPLEMENTARY INFORMATION**

**Supplementary Table 1**: Berlin Sleep Apnoea questionnaire scoring criteria (Chung et al., 2008).

| **CATEGORY 1** | |
| --- | --- |
| Item 1 | If ‘Yes’, assign 1 point |
| Item 2 | If ‘c’ or ‘d’ is the response, assign 1 point |
| Item 3 | If ‘a’ or ‘b’ is the response, assign 1 point |
| Item 4 | If ‘a’ is the response, assign 1 point |
| Item 5 | If ‘a’ or ‘b’ is the response, assign 2 points |
| *Category 1 is positive if the total score is 2 or more points.* | |
| **CATEGORY 2** | |
| Item 6 | If ‘a’ or ‘b’ is the response, assign 1 point |
| Item 7 | If ‘a’ or ‘b’ is the response, assign 1 point |
| Item 8 | If ‘a’ is the response, assign 1 point |
| *Category 2 is positive if the total score is 2 or more points.* | |
| **CATEGORY 3** | |
| *Category 3 is positive if the answer to item 10 is ‘Yes’ or if the BMI of the patient is greater than 30kg/m2.* | |

*Item 9 is noted separately

**Supplementary Table 2**: SF-36 Health Status Survey scoring criteria (Brazier et al., 1992).

| **Item number** | **Original response** | **Recoded value:** |
| --- | --- | --- |
| 18, 20, 32, 34 | Not at all / definitely true → | 100 |
|  | Slightly / a little bit / mostly true → | 75 |
|  | Moderately / don’t know → | 50 |
|  | Quite a bit / Mostly false → | 25 |
|  | Extremely / Definitely false → | 0 |
| 1, 2, 3, 4, 5, 6, 7, 8, 9, 10 | Yes, a lot → | 0 |
|  | Yes, a little → | 50 |
|  | No, not at all → | 100 |
| 11, 12, 13, 14, 15, 16, 17 | Yes → | 0 |
|  | No → | 100 |
| 19, 21, 24, 25, 28 | None / all of the time → | 100 |
|  | Very mild / most of the time → | 80 |
|  | Mild / a good bit of the time → | 60 |
|  | Moderate / some of the time → | 40 |
|  | Severe / a little bit of the time → | 20 |
|  | Very Severe / none of the time → | 0 |
| 22, 23, 26, 27, 29 | All of the time → | 0 |
|  | Most of the time → | 20 |
|  | A good bit of the time → | 40 |
|  | Some of the time → | 60 |
|  | A little bit of the time → | 80 |
|  | None of the time → | 100 |
| 30, 31, 33 | Definitely true / all of the time → | 0 |
|  | Mostly true / most of the time → | 25 |
|  | Don’t know / a good bit of the time → | 50 |
|  | Mostly false / some of the time → | 75 |
|  | Definitely false / a little bit of the time → | 100 |

**Supplementary Table 3**: Australian Recommended Food Score (ARFS) scoring criteria (Collins et al., 2015).

| **Type of food** | **Scoring Criteria** | |
| --- | --- | --- |
| Vegetables  /22 pts | 1pt if 1-5 different vegetables/day  1pt if 1-4 serve of vegetables/day  1pt for each of the following vegetables:   - potatoes - pumpkin - beetroot - cauliflower - green beans - spinach - cabbage - peas - broccoli | - carrots - zucchini - peppers mushroom - fresh tomato - lettuce - celery - cucumber - avocado - onion   2 points if >5 vegetables/day  2 points if >5 serves of vegetables/day  *0 pts overall if <1 different vegetables/day or no serves of vegetables/day* |
| Fruit  /12 pts | 1pt if ≥1 piece of fruit per day,  1 pts if ≥1 of each of the following fruit:   - frozen fruit - apple - pear - citrus | - banana - stone fruit mango - pineapple - berries - apricots - melon   *0 pts if <1 piece of fruit/day* |
| Meat  /7 pts | 1pt for 1-4 serves of   - beef - veal - chicken | - lamb - pork   1 pt for >1 serve Fish (tinned or fresh) |
| Vege protein  /6 pts | 1pt for ≥1 per week of the following:   - nuts (e.g., peanuts, almonds) - nut butters, - eggs, | - soybeans/ tofu, - baked beans,   Other beans or lentils (e.g., chickpeas, split peas). |
| Bread, cereals and grains | 1pt usual bread is   - Rye, high fibre white   2pt if usual bread is multigrain, wholemeal.  1pt ≥1/week for   - Muesli | - Porridge - Cereal - Bread (consumption) - Rice - Wheat cereal - Pasta - All bran cereal |
| Dairy | 1pt ea for ≥2 serves/day of:   - Milk - Yoghurt - Cheese;   1pt for ≥1 serve/week but ≤1 serves/day of;   - Flavoured milk, ice cream, frozen yoghurt; | 1pt for ≥1 serve/week but ≤4 serves/day of;   - cheese, cheese spread or cream cheese;   1pt ea for ≥1 serve/week of   - Plain milk, - Yoghurt (not frozen), cottage cheese or ricotta.   2 points if usual type of milk is reduced fat milk or skim milk, or soy milk |
| Spreads/sauces | 1pt for ≥1 serve per week of:   - Yeast extract spread, tomato or barbecue sauce | |


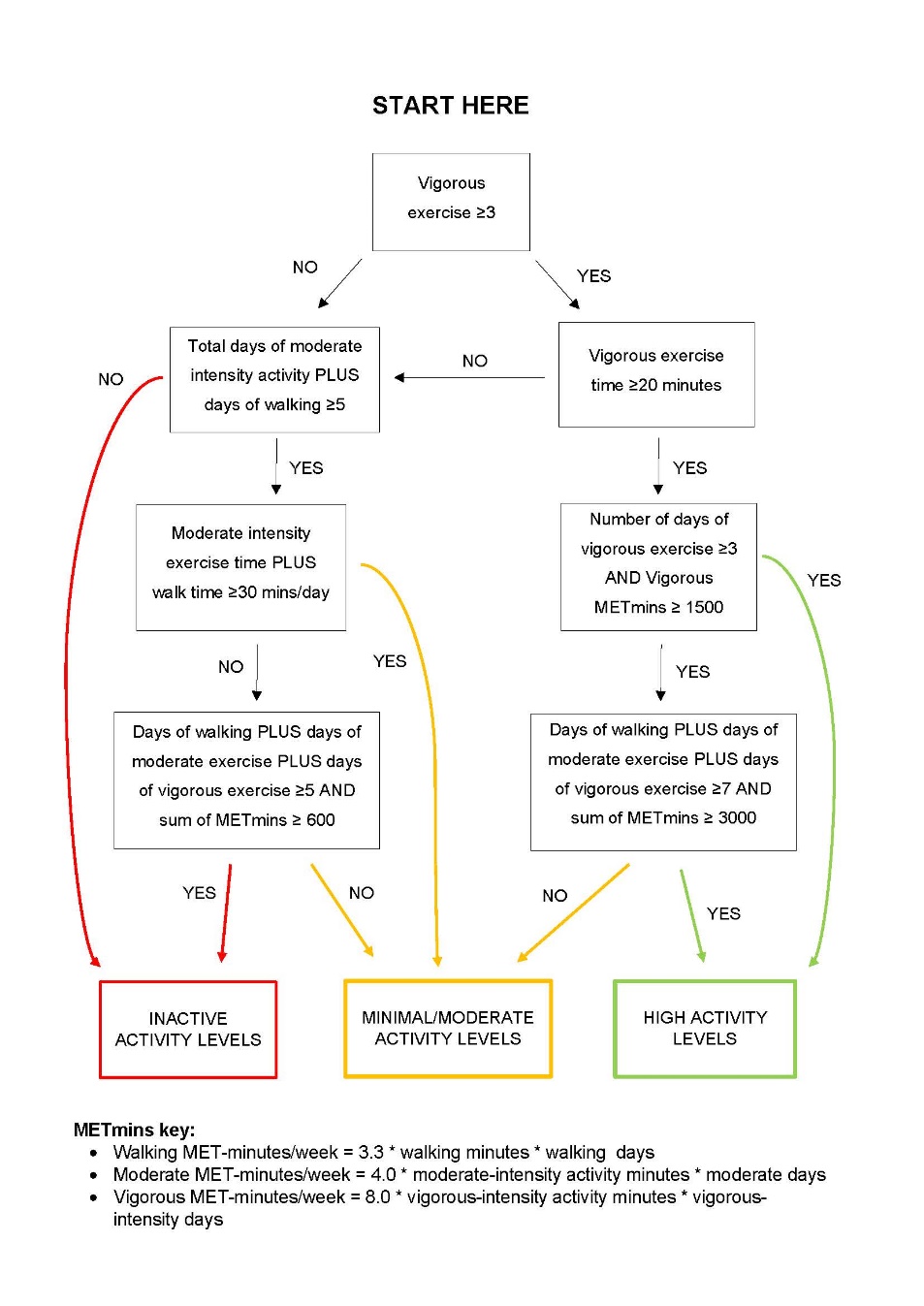


**Supplementary Figure 1**: Scoring of International Physical Activity Questionnaire (IPAQ) (Hagstromer et al., 2006).

**
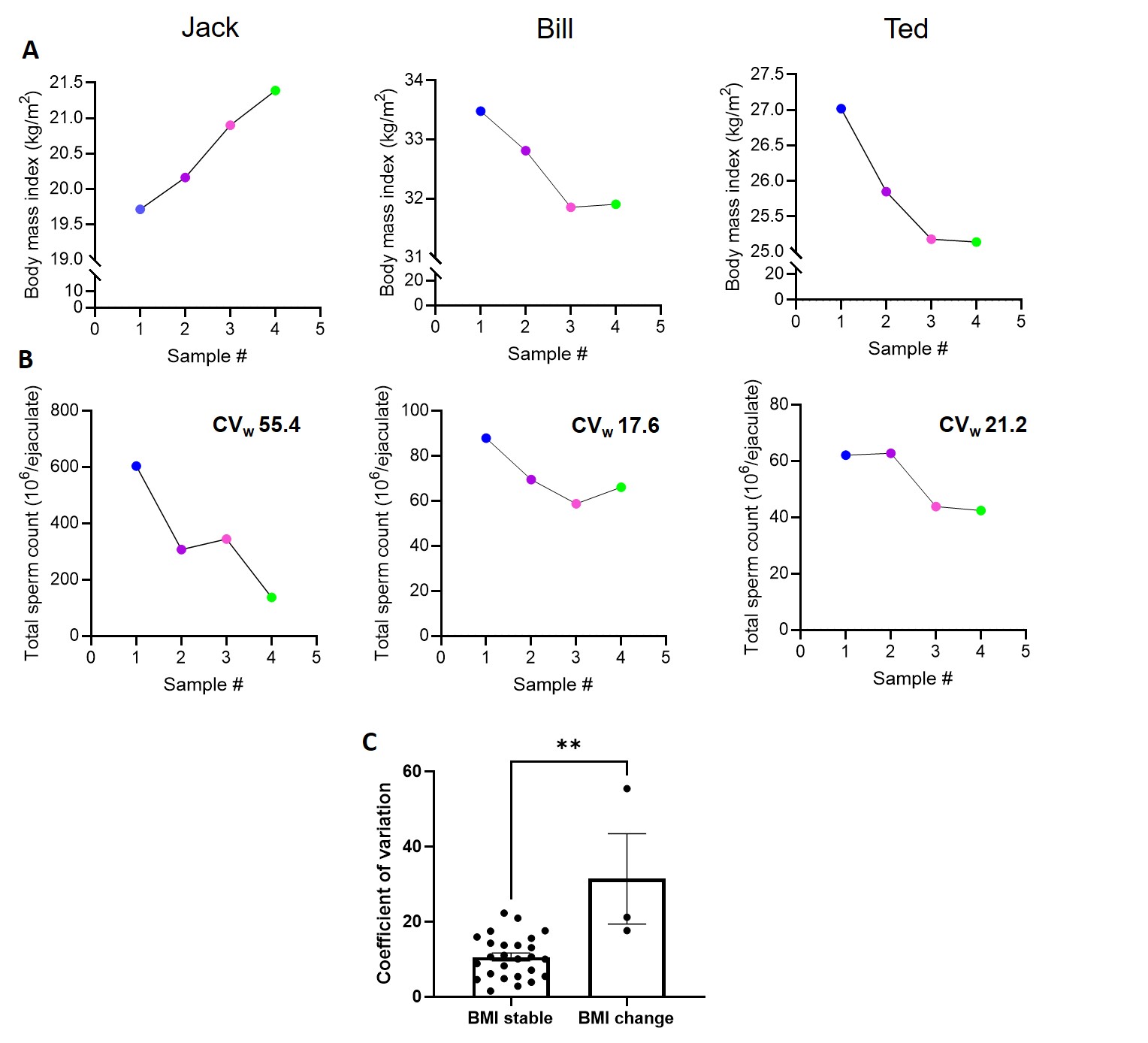
**

**Supplementary Figure 2:** The change in total sperm count in repeat semen collections in those participants who displayed at least a 2kg/m^2^ BMI change.

(A) Change in body mass index (kg/m^2^) at each semen collection, (B) Change in total sperm concentration at each semen collection and (C) Differences in total sperm count coefficient of variation between those men (N=3) who displayed at least a 2kg/m^2^ BMI change over collections compared with men (N=26) whose BMI remained stable. Data was analysed by an unpaired Mann-Whitney U test in untransformed data. **P<0.01. Names are pseudo.
